# Growth medium-dependent antimicrobial activity of early stage MEP pathway inhibitors

**DOI:** 10.1371/journal.pone.0197638

**Published:** 2018-05-17

**Authors:** Sara Sanders, David Bartee, Mackenzie J. Harrison, Paul D. Phillips, Andrew T. Koppisch, Caren L. Freel Meyers

**Affiliations:** 1 Department of Pharmacology and Molecular Sciences, The Johns Hopkins University School of Medicine, Baltimore, MD, United States of America; 2 Department of Chemistry, Northern Arizona University, Flagstaff, AZ, United States of America; Universite Paris-Sud, FRANCE

## Abstract

The *in vivo* microenvironment of bacterial pathogens is often characterized by nutrient limitation. Consequently, conventional rich *in vitro* culture conditions used widely to evaluate antibacterial agents are often poorly predictive of *in vivo* activity, especially for agents targeting metabolic pathways. In one such pathway, the methylerythritol phosphate (MEP) pathway, which is essential for production of isoprenoids in bacterial pathogens, relatively little is known about the influence of growth environment on antibacterial properties of inhibitors targeting enzymes in this pathway. The early steps of the MEP pathway are catalyzed by 1-deoxy-d-xylulose 5-phosphate (DXP) synthase and reductoisomerase (IspC). The in vitro antibacterial efficacy of the DXP synthase inhibitor butylacetylphosphonate (BAP) was recently reported to be strongly dependent upon growth medium, with high potency observed under nutrient limitation and exceedingly weak activity in nutrient-rich conditions. In contrast, the well-known IspC inhibitor fosmidomycin has potent antibacterial activity in nutrient-rich conditions, but to date, its efficacy had not been explored under more relevant nutrient-limited conditions. The goal of this work was to thoroughly characterize the effects of BAP and fosmidomycin on bacterial cells under varied growth conditions. In this work, we show that activities of both inhibitors, alone and in combination, are strongly dependent upon growth medium, with differences in cellular uptake contributing to variance in potency of both agents. Fosmidomycin is dissimilar to BAP in that it displays relatively weaker activity in nutrient-limited compared to nutrient-rich conditions. Interestingly, while it has been generally accepted that fosmidomycin activity depends upon expression of the GlpT transporter, our results indicate for the first time that fosmidomycin can enter cells by an alternative mechanism under nutrient limitation. Finally, we show that the potency and relationship of the BAP-fosmidomycin combination also depends upon the growth medium, revealing a striking loss of BAP-fosmidomycin synergy under nutrient limitation. This change in BAP-fosmidomycin relationship suggests a shift in the metabolic and/or regulatory networks surrounding DXP accompanying the change in growth medium, the understanding of which could significantly impact targeting strategies against this pathway. More generally, our findings emphasize the importance of considering physiologically relevant growth conditions for predicting the antibacterial potential MEP pathway inhibitors and for studies of their intracellular targets.

## Introduction

Studies designed to illuminate the *in vivo* microenvironment of bacterial pathogens during the process of infection have brought to light the poor predictive value of conventional, nutrient-rich *in vitro* culture conditions to broadly examine microbial physiology and evaluate activity of antimicrobial agents [1–3]. Despite the resources available to understand the changes that occur during growth in varied environments and the known disparity between test and physiological growth conditions, the effects of medium composition on antimicrobial activity remain underappreciated. Currently, standard rich growth conditions prevail in antimicrobial discovery efforts.

Growth environment is particularly salient when evaluating agents targeting essential metabolic pathways. Bacterial metabolism is highly regulated in response to environmental conditions and metabolic flexibility is crucial for adaptation to nutrient limiting microenvironments encountered during pathogenesis and infection [4,5]. Nonphysiological conditions may obscure potent inhibitory activity of compounds during antimicrobial screening, particularly inhibitors of metabolic processes that are functional and essential only in the *in vivo* context (cryptic drug targets). This is illustrated by the carbon source dependence of inhibitors of the glyoxylate shunt in gram-negative pathogens [6–8]. Identifying growth condition-dependent hits that lack *in vivo* activity is also problematic. This is exemplified by frequent glycerol-dependent hits from whole cell antitubercular screens conducted in standard glycerol-containing culture conditions which are inactive *in vivo* where glycerol metabolism is not utilized by the pathogen [9–12].

**Fig 1:**
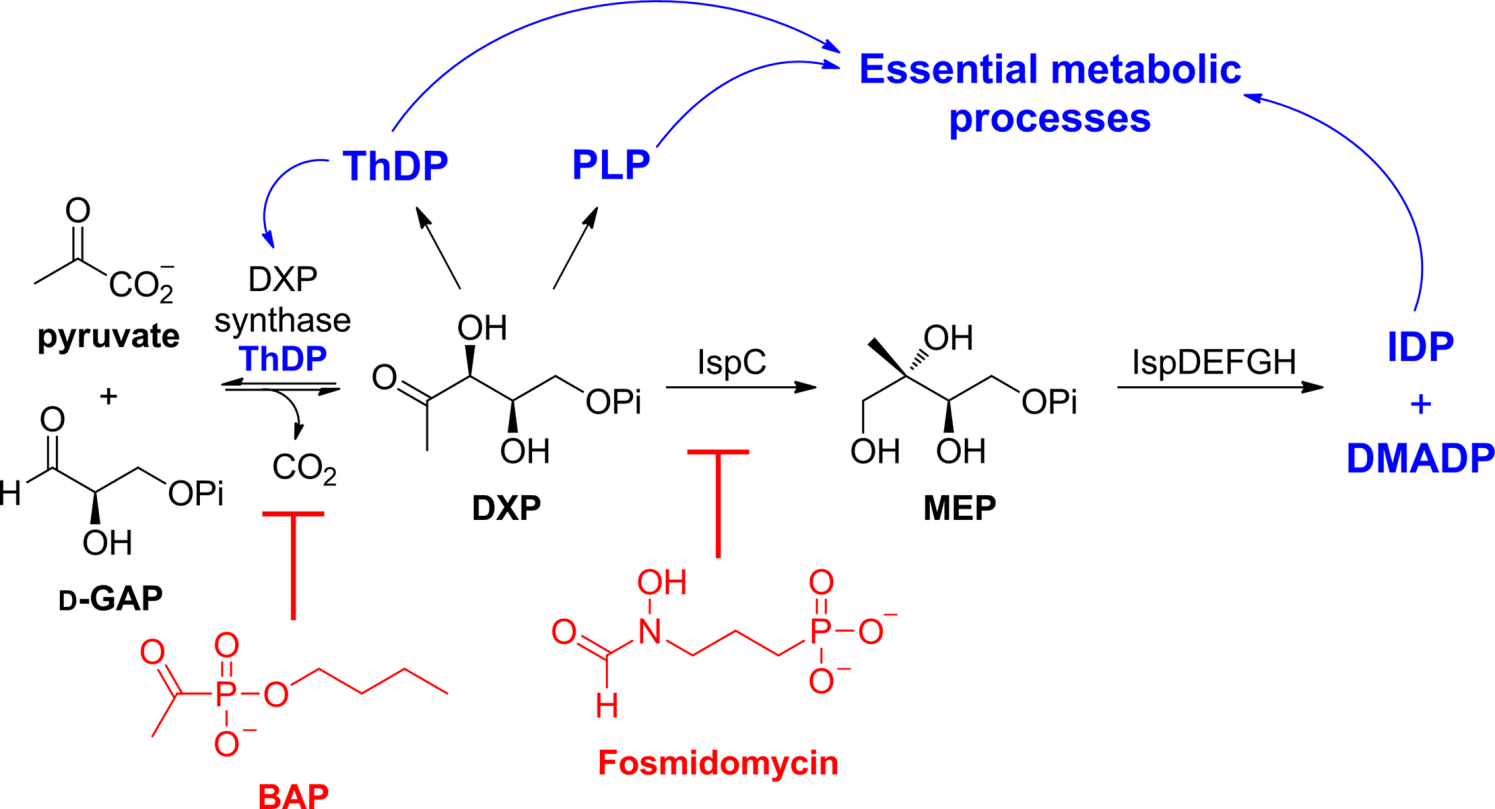
Two early stage MEP pathway inhibitors and their targets are shown in the context of the *E*. *coli* branchpoint metabolite, DXP. Butylacetylphosphonate (BAP) is an inhibitor of DXP synthase, and fosmidomycin is an inhibitor of IspC, the first committed step in isoprenoid biosynthesis. (Pi = PO_4_^2−^).

The methylerythritol phosphate (MEP) pathway (Fig 1) is required for isoprenoid biosynthesis in apicomplexan parasites, plants, and many bacterial pathogens. The pathway is essential for bacterial growth and survival [4,13–19] and for virulence during bacterial infection [15,20,21], making it a potential antimicrobial target [22–32]. However, despite the importance of this pathway within bacterial pathogens, relatively little is known about the influence of growth environment on antibacterial properties of inhibitors targeting this pathway. The early rate-determining steps, catalyzed by 1-deoxy-d-xylulose 5-phosphate (DXP) synthase and DXP reductoisomerase (IspC, MEP synthase), have been studied as potential drug targets.

IspC is the first committed step of the MEP pathway, catalyzing the conversion of DXP to MEP. Fosmidomycin and analogs are potent, selective IspC inhibitors that show potent antimicrobial activity against many Gram-negative bacteria [16,25,29,33,34]. The antimicrobial effects of fosmidomycin have been studied extensively in rich growth medium, however, fosmidomycin activity is not well-studied in nutrient limitation conditions thought to be more relevant to the *in vivo* growth environments of pathogens during infection [35–37].

1-Deoxy-d-xylulose 5-phosphate (DXP) operates immediately upstream of IspC, catalyzing the condensation of pyruvate and glyceraldehyde-3-phosphate to produce DXP. DXP sits at a central metabolic branchpoint (Fig 1), feeding into the biosynthetic pathways for isoprenoids and vitamins thiamin diphosphate (ThDP) and pyridoxal phosphate (PLP), all of which are critical for cell growth and survival [14,32,38–42]. Alkylacetylphosphonates [23,26,43] were designed to selectively inhibit DXP synthase based on its distinct random sequential mechanism [44–47] and large active site volume [48] which, together with its unique domain arrangement [49], distinguish it from other ThDP-dependent enzymes in mammalian and bacterial metabolism [14,32,50]. Butylacetylphosphonate (BAP) displays potent antibacterial activity that depends strongly upon growth medium, with its most potent activity observed under nutrient limitation and minimal activity in rich growth medium.

Intervention at multiple steps in microbial isoprenoid biosynthesis has been studied [23,51–53], and shown in some cases to be synergistic. Representative synergistic combinations within the MEP pathway include BAP and fosmidomycin [23], fosmidomycin in combination with inhibitors targeting farnesyl diphosphate synthase (FPPS) [52], and the synthetic lethality observed when cells are depleted of MEC synthase (2-*C*-methyl-d-erythritol 2,4-cyclodiphosphate synthase, IspF) and treated with fosmidomycin [53]. However, these findings are based upon studies conducted in rich growth medium that likely do not broadly mimic the *in vivo* microenvironment and therefore may not have clinical relevance for all bacterial infections. The recently discovered medium dependence of BAP activity underscores the need to study the growth medium effects on MEP pathway intervention in more depth to better understand the potential of these inhibitors as antibacterial agents.

Herein we present growth medium-dependent activities of BAP and fosmidomycin. Potencies of both inhibitors, alone and in combination, depend strongly upon growth medium, and differences in cellular uptake apparently drive these potency changes. The medium-dependent potency profiles of fosmidomycin and BAP are dissimilar; shifting from rich to minimal medium significantly reduces the potency of fosmidomycin but increases the potency of BAP. While it has been generally accepted that fosmidomycin activity depends upon GlpT transporter expression, our results indicate that fosmidomycin can enter cells by an alternative, less efficient mechanism under nutrient limitation. Finally, the potency and relationship of the BAP-fosmidomycin combination depends upon the growth medium, and a striking loss of BAP-fosmidomycin synergy is observed under nutrient limitation. The change in the BAP-fosmidomycin relationship suggests a shift in the metabolic and/or regulatory networks surrounding DXP accompanying the change in growth medium, the understanding of which could significantly impact targeting strategies against this pathway. This study underscores the significance of considering a broader range of physiologically relevant growth conditions for predicting the antibacterial potential of MEP pathway inhibitors, and for studies of their intracellular targets.

## Results

### BAP is bacteriostatic in M9-glucose growth medium

**Fig 2:**
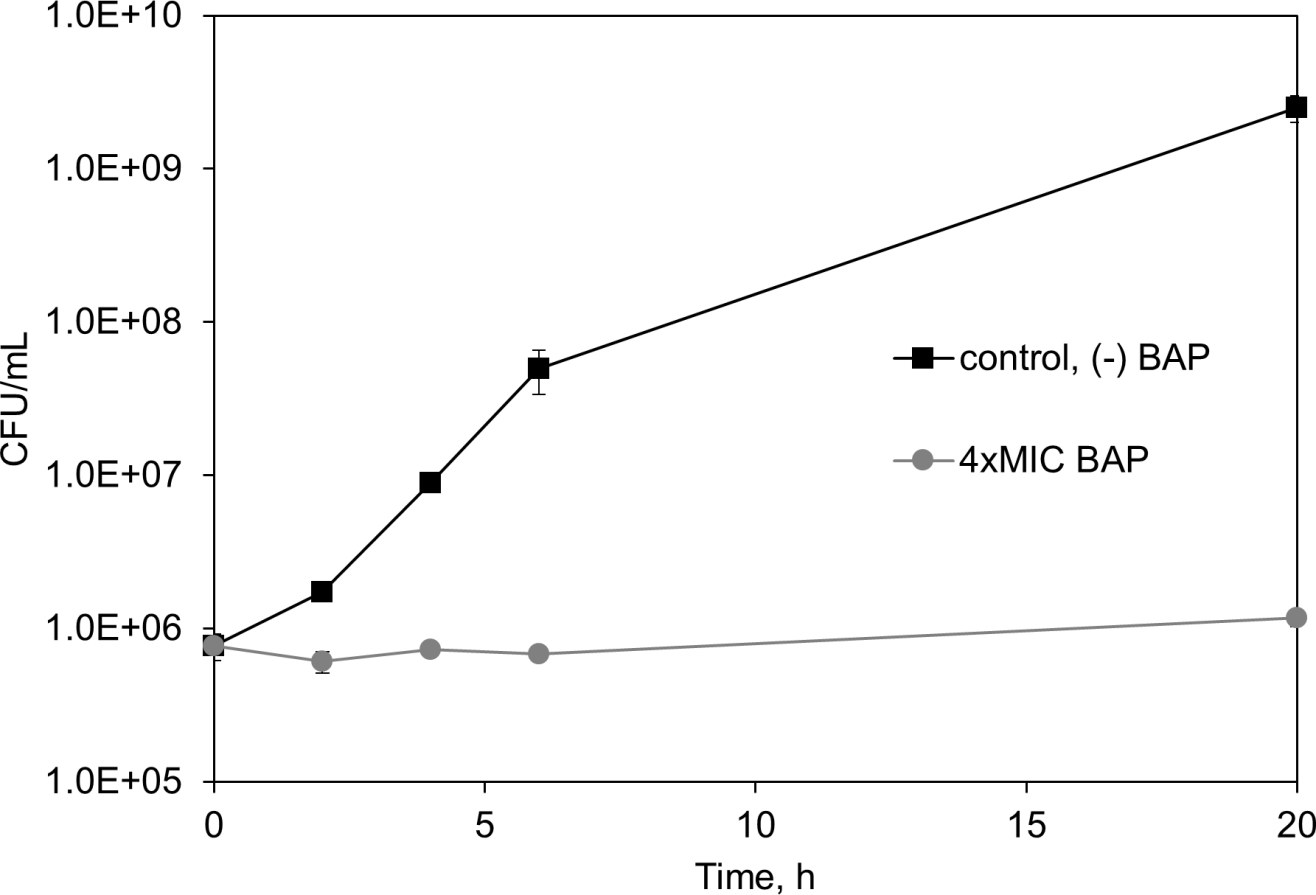
BAP is bacteriostatic. *E*. *coli* cultures at an initial inoculum density of 10^6^ CFU/mL in M9-glucose were incubated in the presence of BAP (●) at 4 × MIC (80 μM, or 15 μg/mL), compared to control in the absence of BAP (■) in biological triplicate. Enumeration of bacteria on agar plates over time (0, 2, 4, 6, and 20 h) indicates that BAP is bacteriostatic. (n = 3, error bars represent standard error).

As noted above, BAP antibacterial potency is highly dependent upon the growth medium, with an MIC_90_ (defined here as the minimal inhibitory concentration required to inhibit 90% of bacterial growth) of 5 μM in M9-glucose minimal medium and exceedingly weak activity under rich growth conditions (CAMHB, [43]). In order to ascertain whether BAP is bacteriostatic or bactericidal under conditions in which it displays greater potency, we performed a time-kill experiment with BAP-treated *E*. *coli* in M9-glucose growth medium. *E*. *coli* cultures at an initial inoculum concentration of 10^6^ CFU/mL were treated with BAP at 4 × MIC (MIC_90_^BAP^ = 20 μM at this cell density) for 20 hours, and bacteria were enumerated on agar plates at various timepoints. The results show that cell density does not decrease over time in the presence of BAP (Fig 2), indicating that BAP is bacteriostatic in M9-glucose minimal medium, with a ratio of MBC (minimal bactericidal concentration) to MIC (MBC/MIC) of at least 4.

### BAP uptake is enhanced under nutrient limitation by an unknown mechanism

**Fig 3:**
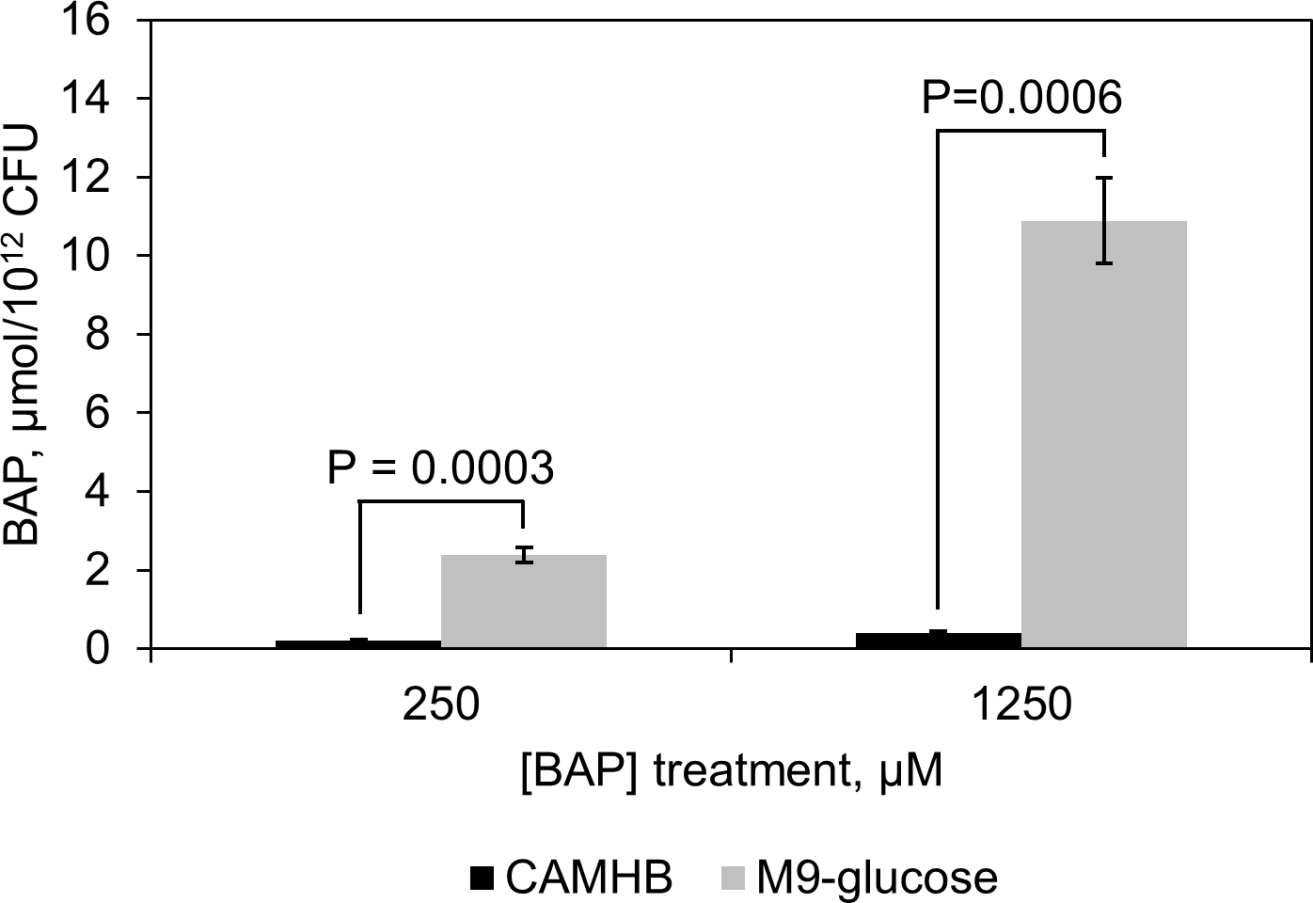
BAP accumulation in *E*. *coli*. *E*. *coli* treated with 250 μM (47 μg/mL) or 1250 μM (233 μg/mL) BAP in CAMHB (■) or M9-glucose (■) medium. Intracellular BAP accumulation was monitored by LC-MS (SRM method). BAP uptake is robust and dose-dependent in M9-glucose medium, and poor in CAMHB medium. (n = 3, error bars are standard error, *p*-values were calculated using an unpaired, 2-sample t-test).

We reasoned that the growth medium-dependent activity of BAP could arise from a difference in the demand for DXP synthase under this growth condition (in the absence of exogenous vitamins and nutrients), or a difference in intracellular BAP concentration in the two media [43]. Under nutrient limitation conditions, uptake efficiency of BAP may be enhanced, possibly due to altered expression of transporters. Thus, intracellular accumulation of BAP in *E*. *coli*, cultured in either M9-glucose minimal or CAMHB medium, was determined by LC-MS (S1A and S1C Fig). Dose-dependent accumulation of BAP is evident in *E*. *coli* grown in M9-glucose at 37°C (Fig 3), compared to a control for non-specific binding of BAP to *E*. *coli* conducted at 0°C (S2 Fig, [54]). Conversely, minimal intracellular accumulation of BAP, slightly above control, is observed in *E*. *coli* grown in rich medium (S2 Fig), approximately 25-fold below the level measured in cells grown in M9-glucose.

The mechanism of entry of BAP into *E*. *coli* grown in M9-glucose minimal medium is unknown. While the bacterial cell wall is largely impenetrable to large molecules, porins enable uptake of small molecules via passive transport in a non-specific manner. The three major porins in *E*. *coli* are OmpC, OmpF, and PhoE, which have been implicated in uptake of antimicrobial compounds [55–64]. OmpA is a minor porin in *E*. *coli*, however, it is upregulated in *E*. *coli* grown in glucose-containing minimal medium and is considered relevant to our studies. As a starting point to explore the mode of entry of BAP into *E*. *coli* in M9-glucose medium, BAP antimicrobial activity was evaluated against a small panel of porin deletion mutants (including Δ*ompA*, Δ*ompC*, Δ*ompF*, and Δ*phoE*). *E*. *coli* deletion mutants lacking each of these porins remain sensitive to BAP, indicating that none of these porins is strictly required for BAP uptake (S3 Fig). However, it is known that porins may compensate for one another; for example, OmpC may compensate for OmpF deletion [65]. Therefore, we evaluated BAP against an *E*. *coli* deletion mutant strain lacking OmpR, required for expression of both OmpC and OmpF [55] (S3 Fig). Minimal shift in BAP potency is observed against this mutant, suggesting OmpC and OmpF may not be required for BAP uptake. However, we cannot definitively rule out porin-mediated uptake given their complex compensatory regulation.

### Activity of the BAP-fosmidomycin combination is bacteriostatic and growth medium-dependent

**Fig 4:**
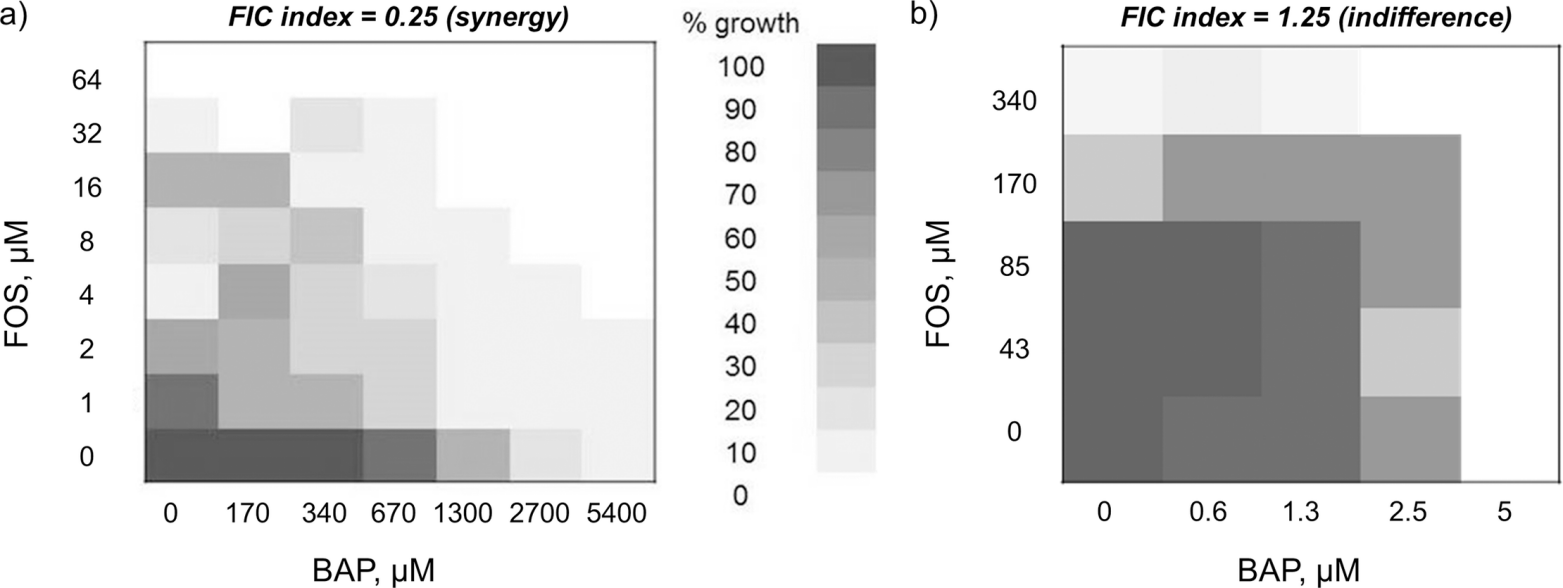
Checkerboard analysis to assess drug interaction between BAP and fosmidomycin. a) Representative heat plot showing a synergistic relationship between BAP (5400 μM, 1000 μg/mL) and fosmidomycin (64 μM, 12 μg/mL) in CAMHB growth medium (figure reproduced with permission, [23]). b) Representative heat plot showing an indifferent relationship between BAP (5 μM, 1 μg/mL) and fosmidomycin (340 μM, 62 μg/mL) in M9-glucose minimal medium with an FIC index range of 1–1.25. The most extreme FIC index value is reported above each heat plot.

Previously, we showed that BAP and fosmidomycin display potent synergistic activity when used in combination against *E*. *coli* grown in rich medium [23]. Considering the dramatic enhancement of BAP activity in M9-glucose minimal medium, we reasoned that this combination could be extraordinarily potent under nutrient limitation. Thus, fosmidomycin was synthesized (S4 Fig, [66,67]), and the activity of BAP in combination with fosmidomycin was evaluated against *E*. *coli* grown in M9-glucose. Interestingly, despite the increased potency of BAP in M9-glucose minimal medium, the synergy between BAP and fosmidomycin is lost in this growth condition, and the relationship between these agents in M9-glucose is indifferent (Fig 4, FIC index ranging from 1 − 1.25). Further, there is an unexpected decrease in the potency of fosmidomycin under nutrient limitation (MIC_90_ = 340 μM, M9-glucose minimal medium), a previously untested growth condition for fosmidomycin activity. The minimum bactericidal concentration (MBC) was determined and analysis of MBC/MIC shows that BAP in combination with fosmidomycin retains a bacteriostatic mechanism under both growth conditions (S5 Fig).

### Medium-dependence of fosmidomycin antimicrobial activity in clinical isolates

Our previous work indicated that conditions of nutrient limitation potentiate BAP against Gram-positive and Gram-negative strains alike. As such, we examined the medium-dependent antibacterial potency of fosmidomycin against a group of clinically isolated bacterial pathogens (Table 1, [68,69,70]). The decrease in potency of fosmidomycin in M9-glucose observed in *E*. *coli* MG1655 is mirrored in a clinical isolate of this species along with several other representative strains. It is worth noting that the strains listed in the table were those that demonstrated robust growth in M9-glucose without exogenous nutrient supplementation (e.g. vitamins, amino acids), which is a necessity in order to screen for this phenomenon.

**Table 1:** Minimum inhibitory concentration (MIC) of fosmidomycin against pathogenic bacteria grown in CAMHB or M9-glucose minimal medium.

	Fosmidomycin MIC_90_, μM (μg/mL)	
Strain	CAMHB	M9-glucose
*Escherichia coli*^*a*^	44 (8)	700 (128)
*Salmonella typhimurium* LT2^*b*^	44 (8)	700 (128)
*Klebsiella oxytoca*^*a*^	87 (16)	1400 (256)
*Klebsiella pneumoniae*^*a*^	350 (64)	>1400 (>256)
*Bacillus thuringiensis* HD 34^*c*^	44 (8)	>1400 (>256)
*Enterobacter cloacae*^*a*^	44 (8)	>1400 (>256)

^*a*^isolate information is available in Leid, et al.

^*b*^ATCC 700720

^*c*^isolate information available in Hill, et al., and Koppisch, et al.

(n = 3, reported values represent the largest of three measured MIC values.)

### Fosmidomycin uptake

**Fig 5:**
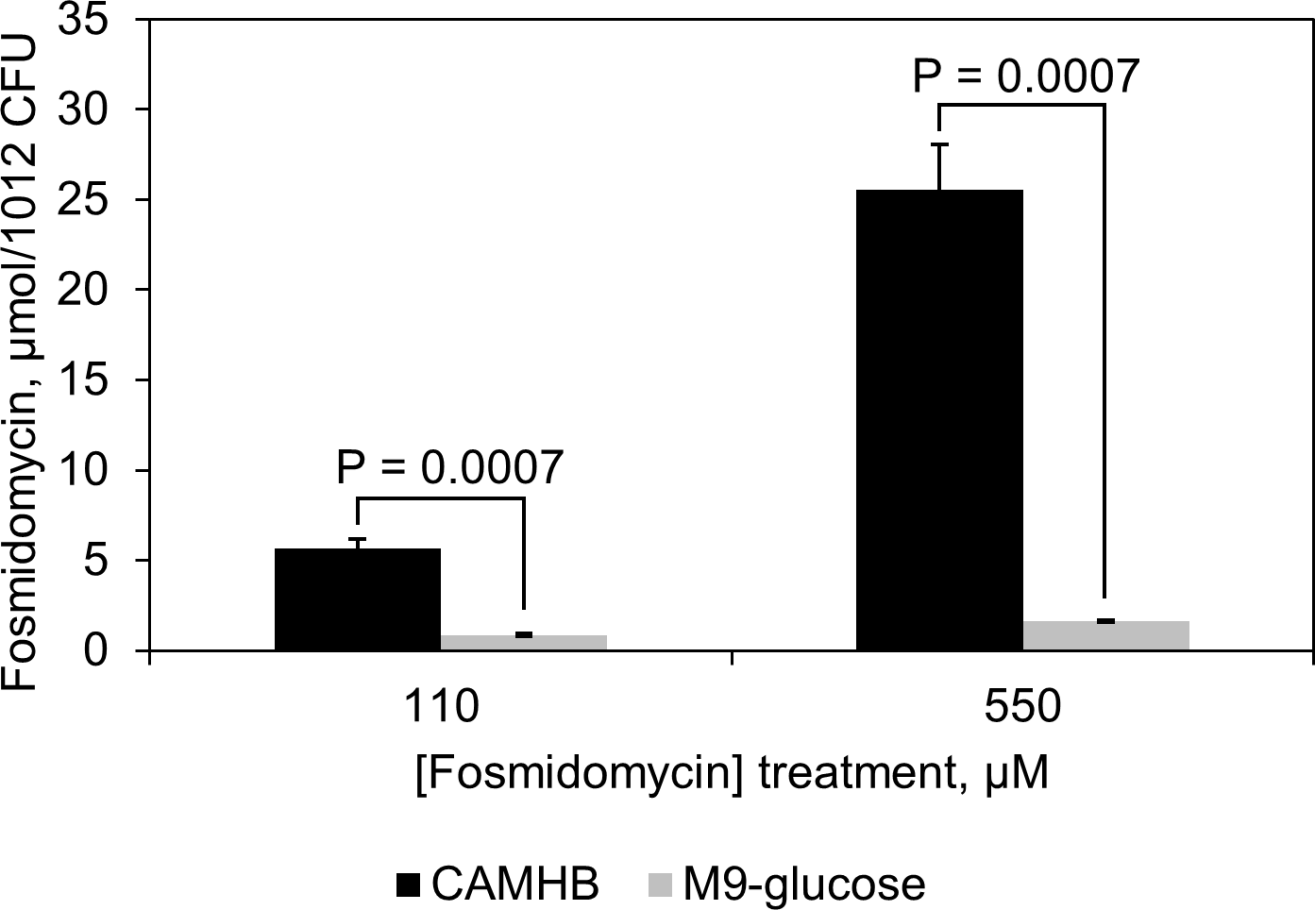
Fosmidomycin accumulation in *E*. *coli*. *E*. *coli* was treated with 110 μM (20 μg/mL) or 550 μM (100 μg/mL) fosmidomycin in CAMHB (■) or M9-glucose (■) medium. Intracellular fosmidomycin accumulation was monitored by LC-MS (SRM method). Fosmidomycin uptake is robust and dose-dependent in CAMHB medium, and poor in M9-glucose medium. (n = 3, error bars are standard error, *p*-values above charts were calculated using an unpaired, 2-sample t-test).

We reasoned that the decreased potency of fosmidomycin against bacteria grown in M9-glucose minimal medium could be a result of lower fosmidomycin accumulation in cells under this condition. To test this hypothesis, an LC-MS uptake assay was developed to detect fosmidomycin levels in *E*. *coli* (S1B and S1D Fig) in varied media. The results indicate that fosmidomycin accumulates to significantly higher levels in cells grown in CAMHB (Fig 5); under the conditions of this assay minimal accumulation is observed in cells grown in M9-glucose (S6 Fig).

The changes in intracellular accumulation of BAP and fosmidomycin in M9 and CAMHB growth media are consistent with the corresponding shifts in potency for BAP and fosmidomycin as single agents under these conditions. However, while BAP uptake appears to be inefficient in *E*. *coli* grown in CAMHB, BAP potency is dramatically enhanced in combination with fosmidomycin under this growth condition. We reasoned that enhanced uptake of BAP in the presence of fosmidomycin could account for this increased potency. Thus, we measured levels of each agent in the presence of the other in CAMHB medium. Interestingly, the accumulation of BAP is not significantly altered in the presence of fosmidomycin (S7A Fig), and fosmidomycin accumulation is only slightly enhanced (25% increase) in the presence of BAP (S7B Fig). Given these results, altered uptake of BAP or fosmidomycin during co-treatment can be excluded as the primary determinant of the potent synergy between these compounds in CAMHB medium.

**Fig 6:**
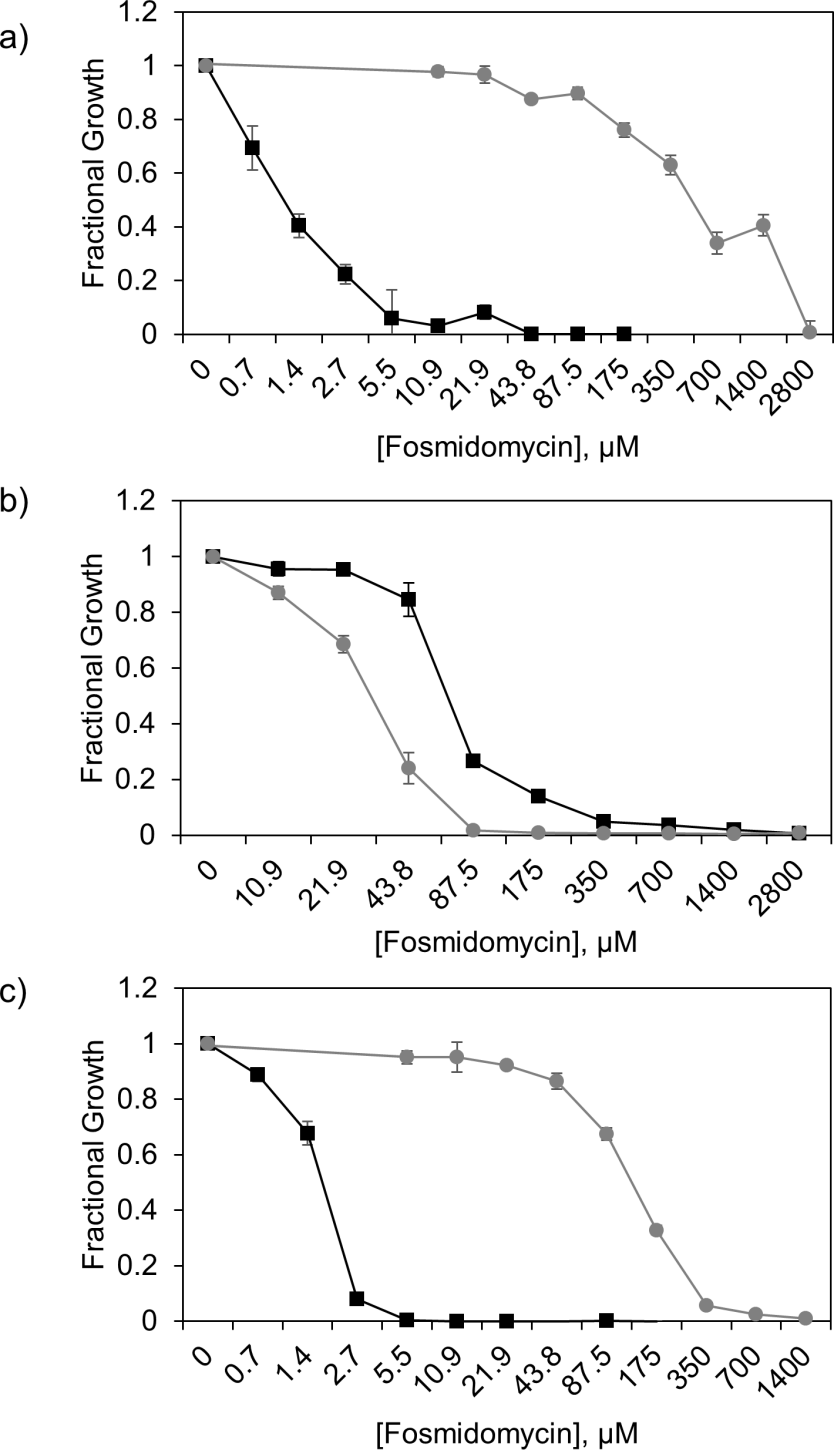
Fosmidomycin antibacterial activity in GlpT deficient *E*. *coli*. GlpT transporter-containing (■ BW25113) and deficient (● Δ*glpT* BW25113) *E*. *coli* strains treated with 2800 μM (512 μg/mL) fosmidomycin in CAMHB (a), M9-glucose (b), and M9-glycerol (c) growth medium. Cell growth was assessed at 16 h (CAMHB, M9-glucose) or 40 h (M9-glycerol) to ensure culture saturation. The large MIC shift between the parent and GlpT deficient strain in CAMHB and M9-glycerol media indicate that GlpT is a fosmidomycin transporter in these growth conditions. (n = 3, error bars represent standard error).

Fosmidomycin is known to enter cells by the glycerol-3-phosphate transporter, GlpT [16,34,71–75]. However, studies of fosmidomycin entry mechanism are largely conducted in rich growth medium, where the effects of GlpT deletion are dramatic (Fig 6A, [16,71,74,76,77]). Pertinent to this study, GlpT is tightly regulated by catabolite repression machinery [78–80] in M9-glucose, and is therefore unlikely to be a major mechanism for fosmidomycin uptake. To further establish that GlpT does not contribute to fosmidomycin uptake under this condition, we determined fosmidomycin activity against the *ΔglpT* strain (Keio Collection, Yale CGSC) in M9-glucose. As expected, fosmidomycin displays similar antibacterial activity (≤ 4-fold shift in MIC_90_) in M9-glucose against the *ΔglpT* strain (MIC_90_ = 88 μM) and the parent *E*. *coli* BW25113 strain (MIC_90_ = 350 μM, Fig 6B). The measurable MIC_90_ and the demonstrated lack of GlpT involvement in fosmidomycin uptake in M9-glucose suggest that fosmidomycin enters *E*. *coli* cells by a distinct mechanism in this growth condition. When the same strains are grown in M9-glycerol minimal medium, fosmidomycin potency decreases against the *ΔglpT* strain compared to the parent strain (MIC_90_^BW25113^ = 3 μM; MIC_90_^Δ;*glpT*^ = 350 μM), suggesting that GlpT is a major transporter for fosmidomycin uptake in M9-glycerol (Fig 6C).

Our results indicate that fosmidomycin potencies against *E*. *coli* lacking GlpT in M9-glucose (MIC_90_^Δ*glpT*^ = 88μM, MIC_90_^BW25113^ = 350 μM) or M9-glycerol (MIC_90_^Δ*glpT*^ = 350 μM) are comparable, suggesting that an alternative mechanism of uptake for fosmidomycin, albeit less efficient than GlpT, exists when cells are cultured minimal medium. As a first step to identify this alternative uptake mechanism for fosmidomycin in M9-glucose, fosmidomycin was evaluated against a small panel of strains lacking *E*. *coli* porins (OmpA, OmpC, OmpF, PhoE) and the porin regulator OmpR. Like BAP, little or no impact on fosmidomycin potency is observed against these deletion mutant strains, indicating uptake by any one of these porins is not strictly required (S3 Fig).

Uptake of a structurally similar antimicrobial agent, fosfomycin, occurs via both GlpT and the glucose-6-phosphate transporter, UhpT, however, UhpT was previously ruled out as a transporter of fosmidomycin in rich medium [76,77,79–82]. UhpT, like GlpT, is regulated by catabolite repression machinery [79–81,83], and is not expected to play a role in fosmidomycin uptake in glucose minimal medium. To establish this definitively and ascertain the role of UhpT in fosmidomycin under nutrient limitation in the absence of catabolite repression, we compared fosmidomycin activity against Δ;*glpT* and parent *E*. *coli* (BW25113) strains in CAMHB, M9-glucose and M9-glycerol. As shown in S8 Fig, fosmidomycin potency is not impacted by deletion of GlpT under these growth conditions, indicating UhpT is not strictly required for fosmidomycin uptake.

## Discussion

While the adoption of standard testing conditions has been necessary and beneficial for clinical diagnostics in infectious disease and comparisons of antibacterial efficacies across research laboratories, antimicrobial drug discovery efforts carried out under standard rich growth conditions may misinform on the antimicrobial activity or altogether fail to identify of inhibitors of metabolic processes. The lack of knowledge regarding the influence of growth environment on antibacterial properties of MEP pathway inhibitors together with our previous work highlighting the interesting growth medium dependence of BAP activity [43] and BAP/fosmidomycin synergy in rich media prompted the current study. Our results show that while BAP retains bacteriostatic behavior alone or in combination with fosmidomycin under the conditions tested, its potency, and the potency of fosmidomycin, are strongly dependent upon growth medium. Fosmidomycin is most potent in rich medium, in contrast to BAP which is most potent under nutrient limitation [43]. These activity profiles are observed across multiple pathogenic Gram-positive and Gram-negative bacterial strains. Differences in intracellular accumulation of each agent appear to underlie the growth medium-dependent activity of each compound, which is ostensibly attributable to varied expression of transporters, including porins, which are also known to be influenced by changes in the cellular environment [5]. While the growth medium-dependent restructuring of cellular metabolism may also contribute to changes in potency of BAP and fosmidomycin, the stark contrast in accumulation of BAP and fosmidomycin in varied media points to uptake as a primary contributor in these cases. Our analysis of BAP and fosmidomycin activity against *E*. *coli* strains lacking the major porins known to mediate antibiotic uptake indicates that OmpC, OmpF, OmpA and PhoE are not absolutely required for uptake of these agents under nutrient limitation. A notable finding from this work is the lack of participation of GlpT in fosmidomycin uptake under nutrient limitation. This represents a departure from the widely-accepted view that transport via GlpT is required for fosmidomycin activity, and together with the observed reduced potency has important implications for fosmidomycin efficacy in nutrient-limited pathogen microenvironments *in vivo*.

Finally, we have revealed an interesting growth medium dependence of the BAP-fosmidomycin relationship, showing a striking loss of synergy of this combination in M9-glucose growth medium. As noted, the medium-dependent uptake of these compounds helps to explain their varying antimicrobial activities in rich and minimal media. However, the loss of synergy of the BAP-fosmidomycin combination in minimal medium cannot be explained by uptake alone. Despite the potential limitations of this uptake assay, including the utilization of a high cell density and early timepoint relative to conditions used in MIC determinations, it is clear that neither BAP nor fosmidomycin uptake in CAMHB is notably enhanced in the presence of the other compound. Rather, it seems likely that the metabolic or regulatory nodes that underlie synergy of the BAP-fosmidomycin combination in rich growth medium are lost with the altered metabolic topology that accompanies growth in M9-glucose minimal medium. Taken together, the results of this study have important implications about the significance of considering pathogen-relevant growth environments to understand and predict antimicrobial effects of agents targeting this metabolic pathway. Investigating medium-dependent activities of MEP pathway inhibitors, alone or in combination, could inform targeting strategies against this pathway *in vivo*.

## Materials and methods

### General methods

Unless otherwise noted, reagents were obtained from commercial sources. Fosmidomycin [66,67] and BAP [26] were synthesized according to previously published methods. Antimicrobial data were collected on a Tecan Infinite M200 Nanoquant plate reader, measuring OD_600_ over time. *E*. *coli* MG1655 was used in antimicrobial and uptake experiments unless otherwise noted. *E*. *coli* Δ;*ompA*, Δ;*ompC*, Δ;*ompF*, Δ;*ompR*, Δ;*phoE*, and Δ;*uhpT* deletion mutants and parent BW25113 strain were obtained from the Yale Coli Genetic Stock Center (New Haven, CT, USA). Clinical isolates of all pathogens were from an in-house strain library maintained at NAU. All microbial manipulation of pathogenic bacteria was conducted in a certified biosafety level 2 laboratory while following all associated safety protocols. The usage of the term MIC_90_ in this manuscript refers to the minimal inhibitory concentration required for 90% inhibition of bacterial growth relative to growth of the cells in the absence of inhibitor. MIC values represent the highest determined from three biological replicates in cases where MICs varied by 2-fold. LC-MS samples were processed using a Waters ACQUITY UPLC column (HSS C18 1.8 μm, 1 × 50 mm) and either a Waters NanoAcquity UPLC and a TSQ Vantage mass spectrometer or an Agilent UHPLC and QToF mass spectrometer.

### Fosmidomycin synthesis

The synthesis of fosmidomycin was performed as described previously [67]. The last step of the synthesis is described below with purification by recrystallization as previously described [66].

Diethyl (N-formy-3-hydroxyamino-propanyl)phosphonate (3.00 g, 12.5 mmol) was dissolved in anhydrous dichloromethane (40 mL) and cooled to 0°C. TMS-Br (13.2 mL, 100 mol) was added dropwise and a light pink color formed. The solution temperature was allowed to warm to 22°C. A light purple color appeared over time. After stirring for 16 h, the solution was a faint tan color. Volatiles were removed under reduced pressure. Acetonitrile was added and then removed under reduced pressure to yield a residue. Water (50 mL) was added to the residue at 0°C and the solution was stirred at 22°C for 1 h. This solution was adjusted to pH 4.8 with 1 M NaOH. Water was removed under reduced pressure and the residue was dissolved in methanol (50 mL) and heated to 60°C. Ethanol (10 mL) was added to give an initial white precipitate, which was later characterized as a deformylated byproduct. After removal of the solids, ethanol (50 mL) was added to precipitate monosodium fosmidomycin. The desired product was collected by vacuum filtration, washed with cold ethanol, and dried *in vacuo* to yield of monosodium fosmidomycin (1.03 g, 40% yield). Monosodium fosmidomycin exists as 2 observable rotamers in an 8:2 ratio. ^1^H NMR (500 MHz, D_2_O) δ 8.24 (s, 0.2H), 7.90 (s, 0.8H), 3.48–3.60 (m, 2H), 1.72–1.93 (m, 1H), 1.42–1.56 (m, 2H). ^31^P NMR (262 MHz, D_2_O, referenced to triphenylphosphine oxide at 0 ppm) δ 0.04 (s, 0.2 P), -0.103 (s, 0.8 P).

### General methods for *E*. *coli* antimicrobial susceptibility to BAP and fosmidomycin

Using aseptic techniques, 3 individual colonies were selected from a plate containing the desired *E*. *coli* strain and inoculated into either Mueller Hinton Broth 2 (CAMHB, containing acid hydrolysate of casein, beef extract, and starch, pH 7.3; Sigma, St. Louis, MO, USA), or M9-glucose minimal medium (containing potassium phosphate, sodium phosphate, sodium chloride, ammonium chloride, magnesium sulfate, calcium chloride, and 0.4% w/v glucose [84], adding 20 μM FeSO_4_ immediately prior to use). Strains from the Keio collection were grown in the presence of Kanamycin due to the Kan^R^ marker utilized in this transposon mutagenesis collection. Inoculated cultures were grown to saturation overnight with shaking at 37°C. The saturated cultures were then subcultured (1:50 dilution) into fresh growth medium and grown to exponential phase as measured by absorbance (OD_600_ = 0.4, [35,43]). Cell cultures at exponential phase were diluted 1:1000 into fresh medium to yield the experimental inoculum which was mixed 1:1 with M9-glucose containing the antimicrobial agent at 2× the desired concentration. The final concentration of bacteria in each well was approximately 10^5^ CFU/mL in a final volume of 200 μL. Colony counts of the experimental inoculum were independently verified by dilution and enumeration on CAMHB agar for 16 h at 37°C to confirm consistency between experiments. The 96-well plates were incubated at 37°C for 16 h with intermittent shaking. Fractional growth of drug-treated cells was determined at 16 h relative to the no drug control. Experiments were performed in triplicate.

### General methods to assess antimicrobial susceptibility of clinical pathogens to BAP and fosmidomycin

The methods to assess potency of BAP or fosmidomycin against the clinical isolates largely parallel those outlined above. In brief, all clinical isolates were streaked onto CAMHB agar and allowed to grow for 12 hr at 37°C. Colonies were inoculated via aseptic technique into CAMHB (5 mL) and grown to saturation overnight, at which time they were subcultured into fresh CAMHB for immediate analysis via the broth microdilution assay (as stated above) or they were subcultured into M9-glucose (approximately 2 μL per 5 mL of fresh medium) and grown to saturation at 37°C overnight. Confluent cultures of the strains in M9-glucose were then subcultured into fresh M9-glucose a second time prior to analysis via broth microdilution assays. All experimental inocula were verified with dilution and enumeration prior to use and at minimum, all experiments were performed in triplicate.

### Checkerboard analysis of BAP and fosmidomycin

An initial inoculum of 10^5^ CFU/mL *E*. *coli* was prepared as described above for M9-glucose minimal medium, and as described by Smith, *et*. *al*. for CAMHB medium [23]. Cells were combined with varied BAP and fosmidomycin (CAMHB: 0–5400 μM BAP, 0–64 μM fosmidomycin; M9-glucose: 0–5 μM BAP, 0–340 μM fosmidomycin) in a checkerboard pattern in a 96-well plate and incubated at 37°C for 16 h. Cell growth was measured by absorbance, and fractional growth was calculated relative to the no drug control. FIC indices were determined as previously described with minor modifications [23]. Briefly, FIC indices were calculated using Eq 1 [85,86].

$$\frac{\left(BAP\right)}{\left({MIC}_{BAP}\right)}+\frac{\left(FOS\right)}{\left({MIC}_{FOS}\right)}={FIC}_{BAP}+{FIC}_{FOS}=FIC\;index\;(FICI)$$ (1)

MIC_BAP_ and MIC_FOS_ represent the lowest concentrations of BAP or FOS (fosmidomycin) showing < 10% growth. FIC_BAP_ was calculated as the [BAP in the presence of FOS] for a well showing < 10% growth, divided by MIC_BAP_. FIC_FOS_ was calculated as the [FOS in the presence of BAP] in the same well, divided by MIC_FOS_. The FIC index is the sum of FIC_BAP_ and FIC_FOS_. FIC indices were used to indicate drug synergism (x < 0.5), additivity (0.5 < x < 1.0), indifference (1.0 < x < 2.0), or antagonism (x > 2). Fractional growth was determined relative to the no drug growth control and average values were used.

### MBC/MIC determination

As described above, an initial inoculum of 10^5^ CFU/mL MG1655 *E*. *coli* was prepared by subculturing saturated overnight cultures, then treated with BAP or BAP and fosmidomycin in combination, in either CAMHB or M9-glucose medium (see checkerboard analysis). After 20 h of growth at 37°C, 1 μL aliquots of cell culture from each well of the 96-well plate were spotted onto agar plates containing the corresponding medium. Colony formation at 1–8 × MIC concentrations indicates a bacteriostatic mechanism of action, while a lack of colony formation indicates some level of bactericidal activity. MBC was defined as the concentration of compound required to kill bacteria, leading to an absence of colonies when spotted on agar plates.

### Sample preparation for uptake analysis by LC-MS

A protocol modified from Zhou, *et*. *al*. and Richter, *et*. *al*. was used for cell sample collection and cell lysis [54,87]. Experiments were performed in biological triplicate. Saturated overnight cultures of MG1655 *E*. *coli* were prepared as described above, and sub-cultured at a dilution of 1:50 into 200 mL of fresh medium. Cultures were incubated with shaking (250 rpm) at 37°C for approximately 3 hours, until an OD_600_ of 0.5–0.7 was reached. Cells were isolated via centrifugation in 4 × 50 mL Falcon tubes at 3220 rpm for 20 minutes at 4°C. After discarding the supernatant, the cell pellets were combined and resuspended in fresh media to a final volume of 8.5 mL. Cells were equilibrated to the treatment temperature (0 or 37°C) for 10 minutes, then treated with BAP or fosmidomycin and incubated for 60 minutes, during which no additional cell growth was noted (by colony count or OD_600_ measurements, data not shown).

At the indicated timepoints, 800 μL of culture was gently suspended onto 500 μL of a 3:1 mixture of silicone oil (Sigma Aldrich, 146153) and dichloromethane in a 1.7 mL microcentrifuge tube. The cells were immediately pelleted through the oil mixture at 12,000 × g to create a density gradient separating cells from the inhibitor-containing medium. After wicking away the aqueous medium with a Kimwipe, tubes were inverted and rested for 5 minutes to allow the oil to drain from the pellet surface. Tubes were cut with a plastic-tubing cutter (Bel-Art SP Scienceware, H21010) at the 500 μL mark, and the cell pellet was transferred to a fresh tube in 2 × 100 μL aliquots of ddH_2_O.

Cell lysis was accomplished following three freeze-thaw cycles in which samples were placed in a liquid nitrogen bath for 3 minutes followed by incubation for 3 minutes in a water bath heated to 65°C, and vortexing between each cycle. Cell debris was pelleted at 12,000 × g for 2 minutes, and 180 μL of supernatant was removed into a fresh tube. Methanol (100 μL) was added to the pellet of cell debris, and the tube was agitated and vortexed before pelleting again at 12,000 × g for 2 minutes. The methanol supernatant (100 μL) was added to the aqueous supernatant to a total volume of 280 μL, and the remaining cell debris was discarded. Combined supernatant samples were vortexed and centrifuged at 16,000 × g for 10 minutes to pellet any protein or debris remaining in the sample. An aliquot of this clarified supernatant was then transferred to an LC-MS vial for analysis. LC-MS methods are detailed in Supplementary Information.

### LC-MS analysis

#### Selective reaction monitoring (SRM) method

Samples were analyzed with an LC-MS/MS system comprised of a Waters NanoAcquity UPLC and a TSQ Vantage Triple Quadripole (Thermo Scientific). The liquid chromatography separation was performed on a Waters Acquity UPLC HSS C18 column (1.0×50 mm, 1.8 μm) with mobile phase A (0.1% triethylammonium acetate in water) and mobile phase B (acetonitrile). The flow rate was 50 μL min^-1^. The autosampler temperature was set at 10 °C. The injection volume was 5 μL. Mass spectra were acquired with negative electrospray ionization at the ion spray voltage of -3,000 V. The capillary temperature was 270°C.

For BAP, the LC gradient was as follows: 0–1 min, 0% B; 1–7 min, 0–100% B; 7–9 min, 00% B; 9–10.1 min, 100–0% B; 10.1–12 min, 0% B. Selective reaction monitoring followed the transition from parent ion to a metaphosphate fragment (179 to 62.9 m/z) using a collision energy of 38 eV.

For fosmidomycin, the LC gradient was as follows: 0–2 min, 0% B; 2–7 min, 0–20% B; 7–7.1 min, 20–100% B; 7.1–9 min, 100% B; 9–10.1 min, 100–0% B; 10.1–12 min, 0% B. Selective reaction monitoring followed the transition from parent ion to a metaphosphate fragment (182 to 79.0 m/z) using a collision energy of 41 eV.

#### Quadripole Time-of-Flight (Q-TOF) method

Samples were analyzed with an LC-MS system comprised of an Agilent 1290 UHPLC and 6540 Q-TOF mass spectrometer with Jet Stream Electrospray Ionization source. The liquid chromatography separation was performed on a Waters Acquity UPLC HSS C18 column (1.0×50 mm, 1.8 μm) with mobile phase A (0.1% triethylammonium acetate in water) and mobile phase B (acetonitrile). The flow rate was 200 μL min^-1^. The autosampler temperature was set at 20°C. The injection volume was 2 μL for BAP and 1 μL for fosmidomycin. The Jet stream ESI source parameters were as follows: Negative Ion Mode, Drying Gas Temp: 350°C; Sheath Gas Temp: 400°C; Drying and Sheath Gas Flow: 12 L min^-1^; Nebulizer: 45 psig; VCap: -3000 V, Nozzle: -600 V, Fragmentor; -100 V; Skimmer: -50 V, OCT 1 RF Vpp: -750 V. The acquisition was performed by full scan MS from m/z 50 to m/z 500. (BAP = 179.0479 m/z; fosmidomycin = 182.0224 m/z)

The LC gradient for BAP was as follows: 0–3 min, 0–100% B; 3–3.2 min, 100% B; 3.21 min, 0% B. For fosmidomycin, the LC gradient was as follows: 0–3 min, 0–50% B; 3–3.5 min, 50–100% B; 3.5–3.7 min, 100% B; 3.71 min. Analyte peaks areas were determined from extracted ion chromatograms from the full scan data with a ± 20 ppm window for BAP and a ± 10 ppm window for fosmidomycin using Agilent Masshunter Quantitative Analysis Software (B.06.00 SP01).

## Supporting information

S1 Fig Standard curves for LC-MS methods. Standard curves of BAP (a, c) and fosmidomycin (b, d) were generated using either the Selective Reaction Monitoring (SRM) method (a, b) or the Quadripole Time-of-Flight (Q-TOF) method (c, d).
(TIF)

S2 Fig BAP accumulation in *E*. *coli* at varied temperature. *E*. *coli* was treated with BAP (1250 μM) for one hour at either 37°C (black square) or 0°C (gray square) in either CAMHB or M9-glucose medium. Intracellular BAP accumulation was monitored by LC-MS (SRM method). (n = 3, error bars represent standard error, *p*-values were calculated using a paired, 2-sample t-test).
(TIF)

S3 Fig Antibacterial activity against porin deletion strains. MIC values were determined in biological triplicate for *E*. *coli* strains lacking bacterial porins (Keio collection: parent BW25113 (dark blue), Δ*ompR* (green), Δ*ompA* (orange), Δ*ompC* (gray), Δ*ompF* (yellow), Δ*phoE* (light blue)) treated with BAP (a) or Fosmidomycin (b) after growth in M9-glucose minimal medium for 16 h. (n = 3, error bars represenet standard error).
(TIF)

S4 Fig Synthesis of fosmidomycin. The synthesis of fosmidomycin was characterized by ^1^H (a) and ^31^P (b) NMR. The TPPO standard has a chemical shift in between that of the two observable rotomeric species.
(TIF)

S5 Fig Bacteriostatic activity of BAP and BAP-fosmidomycin. *E*. *coli* cultures were grown in M9-glucose minimal medium (a) or CAMHB rich medium (b) in 96-well plates for 20 hours. Cell cultures (1 μL per well) were spotted onto agar plates containing the corresponding medium, incubated, and imaged. Red asterisks (*) indicate the MIC (fractional growth of 10% or less relative to the no drug control) of representative replicates. The BAP-fosmidomycin combination is bacteriostatic (c), indicated by an MBC/MIC ≥ 8.
(TIF)

S6 FigFosmidomycin accumulation in *E*. *coli* at varied temperature.*E*. *coli* was treated with 550 μM (100 μg/mL) fosmidomycin for one hour at either 37°C (■) or 0°C (■) in either M9-glucose or CAMHB medium. Intracellular fosmidomycin accumulation was monitored by LC-MS (Q-TOF method). (n = 3, error bars represent standard error, *p*-values were calculated using an unpaired, 2-sample t-test).(TIF)

S7 Fig Accumulation in *E*. *coli* of BAP or fosmidomycin alone and in combination. *E*. *coli* was treated with 550 μM (100 μg/mL) fosmidomycin, 1250 μM (230 μg/mL) BAP, or both for one hour in CAMHB growth medium. Intracellular BAP (a) and fosmidomycin (b) accumulation was monitored by LC-MS (Q-TOF method). (n = 3, error bars represent standard error, *p*-values above charts were calculated using an unpaired, 2-sample t-test).
(TIF)

S8 FigAntibacterial activity of fosmidomycin against UhpT deficient *E*. *coli*.UhpT transporter-containing (■ BW25113) and deficient (▲ Δ*uhpT* BW25113) *E*. *coli* strains were treated with fosmidomycin in CAMHB (a), M9-glucose (b), and M9-glycerol (c) growth medium in biological triplicate. Deletion of UhpT does not significantly impact susceptibility to fosmidomycin.(TIF)
