# Supplementary figures and images for: Growth medium-dependent antimicrobial activity of early stage MEP pathway inhibitors
Source: PLoS One. 2018 May 17;13(5):e0197638. doi: 10.1371/journal.pone.0197638 (PMC5957436; doi:10.1371/journal.pone.0197638)

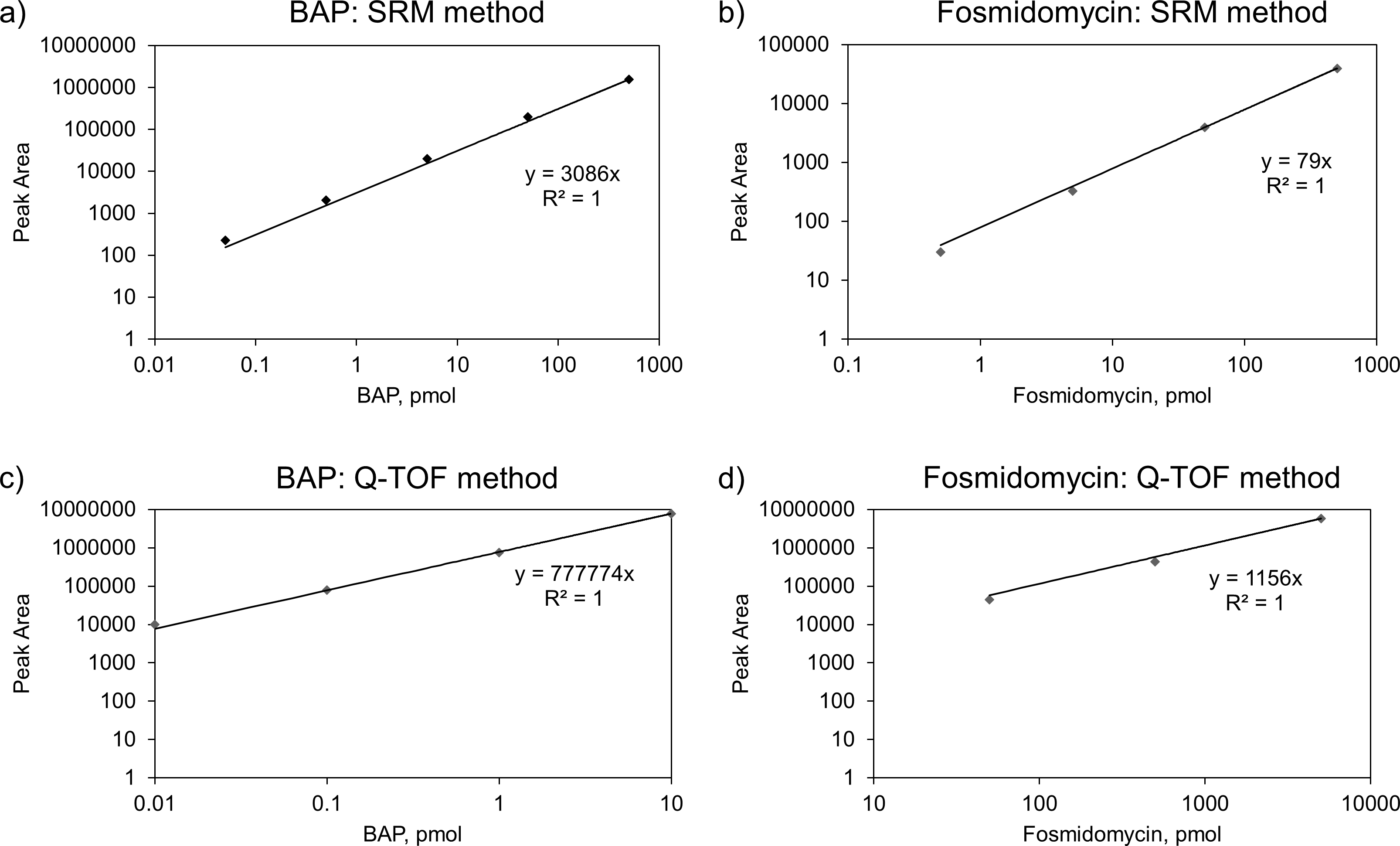

Supplement: S1 Fig — Standard curves of BAP (a, c) and fosmidomycin (b, d) were generated using either the Selective Reaction Monitoring (SRM) method (a, b) or the Quadripole Time-of-Flight (Q-TOF) method (c, d). (TIF) [file pone.0197638.s001.TIF]

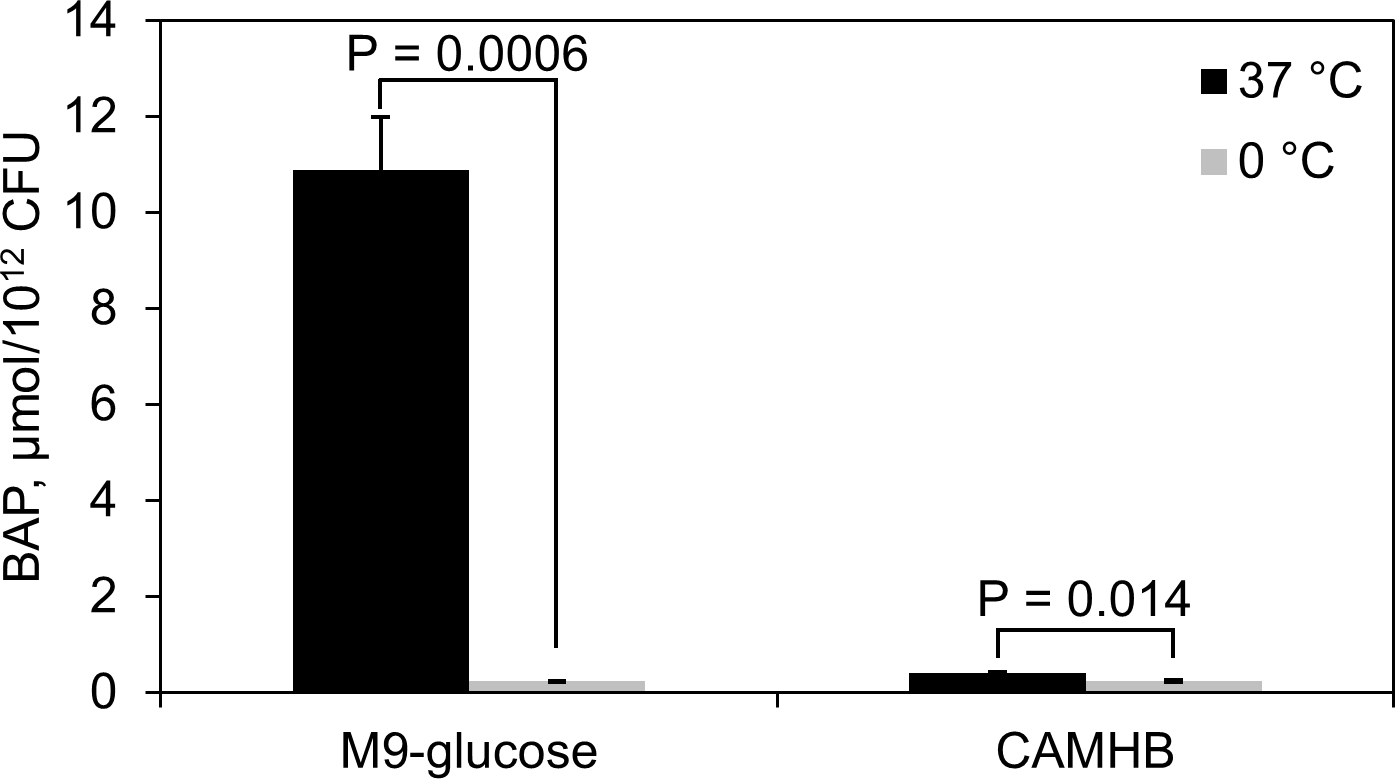

Supplement: S2 Fig — E. coli was treated with BAP (1250 μM) for one hour at either 37°C (black square) or 0°C (gray square) in either CAMHB or M9-glucose medium. Intracellular BAP accumulation was monitored by LC-MS (SRM method). (n = 3, error bars represent standard error, p-values were calculated using a paired, 2-sample t-test). (TIF) [file pone.0197638.s002.TIF]

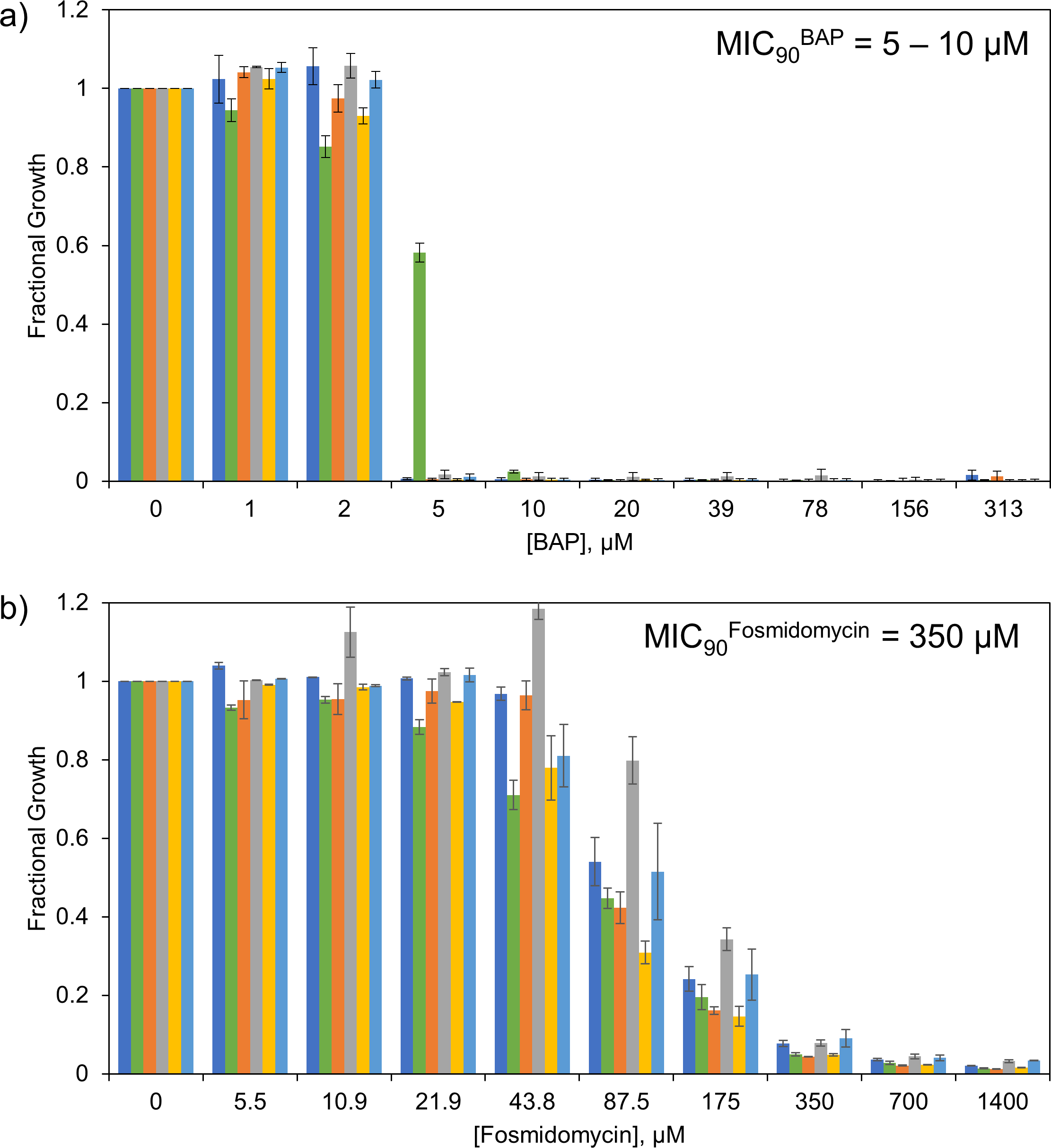

Supplement: S3 Fig — MIC values were determined in biological triplicate for E. coli strains lacking bacterial porins (Keio collection: parent BW25113 (dark blue), ΔompR (green), ΔompA (orange), ΔompC (gray), ΔompF (yellow), ΔphoE (light blue)) treated with BAP (a) or Fosmidomycin (b) after growth in M9-glucose minimal medium for 16 h. (n = 3, error bars represenet standard error). (TIF) [file pone.0197638.s003.TIF]

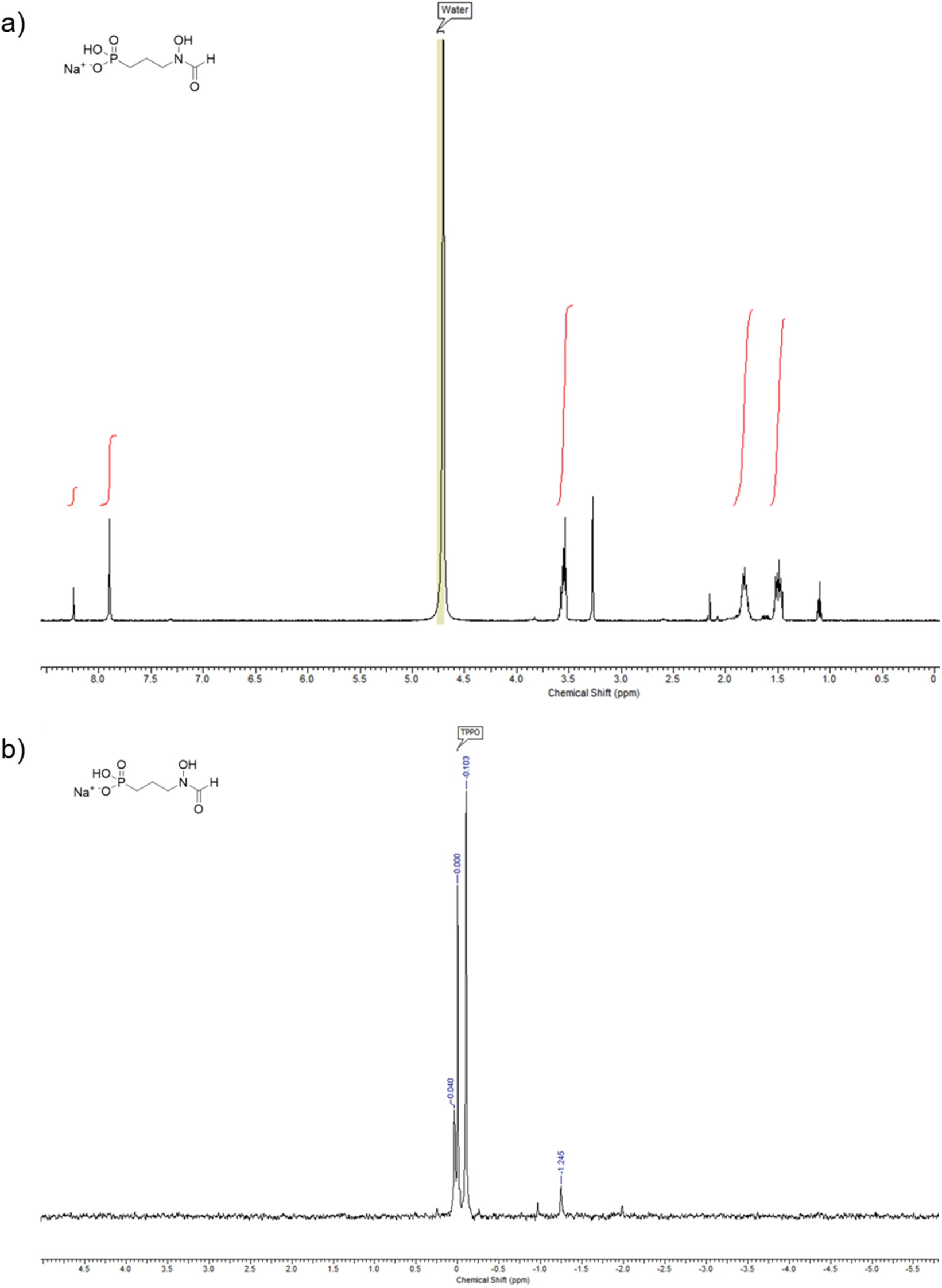

Supplement: S4 Fig — The synthesis of fosmidomycin was characterized by 1H (a) and 31P (b) NMR. The TPPO standard has a chemical shift in between that of the two observable rotomeric species. (TIF) [file pone.0197638.s004.tif]

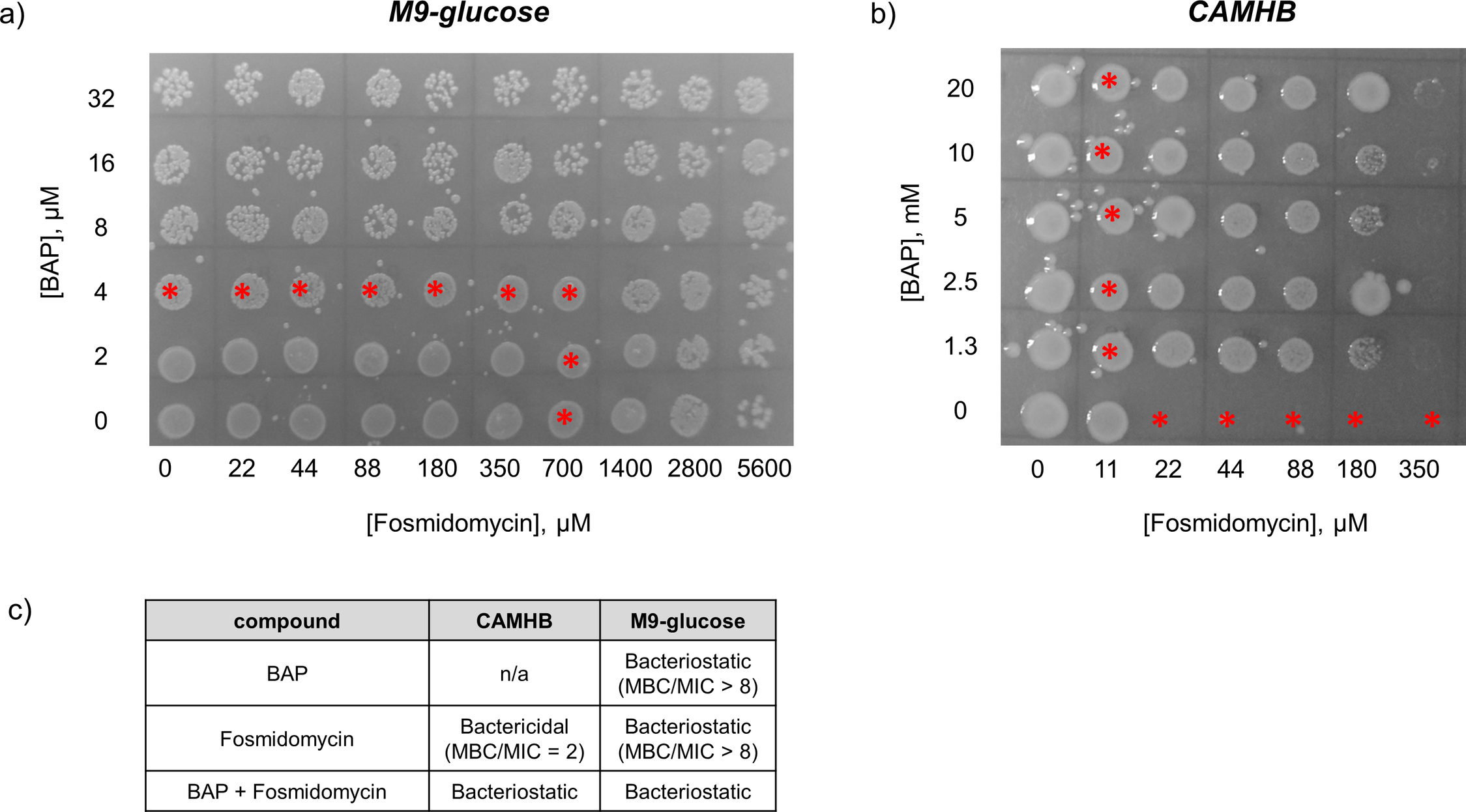

Supplement: S5 Fig — E. coli cultures were grown in M9-glucose minimal medium (a) or CAMHB rich medium (b) in 96-well plates for 20 hours. Cell cultures (1 μL per well) were spotted onto agar plates containing the corresponding medium, incubated, and imaged. Red asterisks (*) indicate the MIC (fractional growth of 10% or less relative to the no drug control) of representative replicates. The BAP-fosmidomycin combination is bacteriostatic (c), indicated by an MBC/MIC ≥ 8. (TIF) [file pone.0197638.s005.tif]

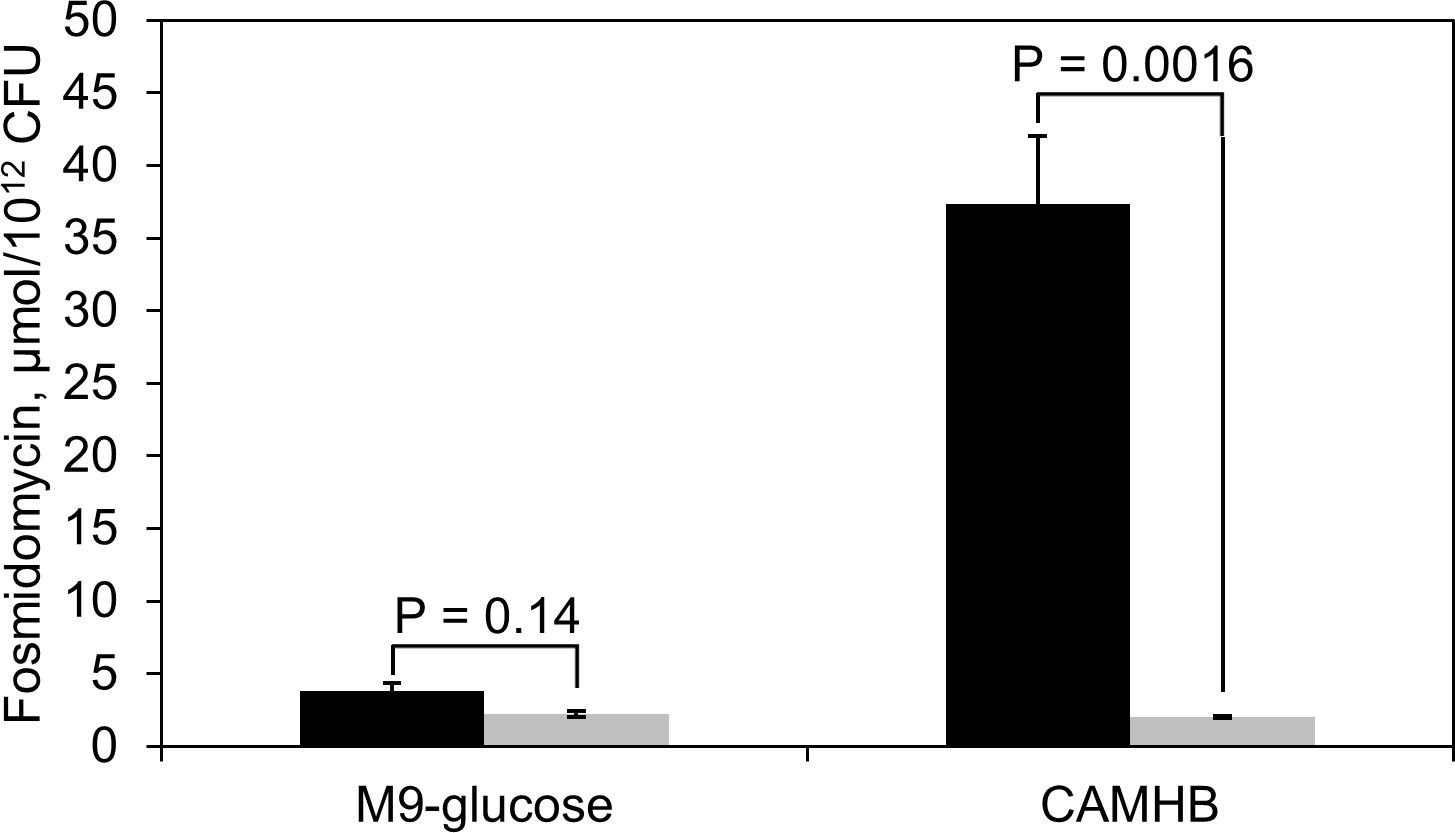

Supplement: S6 Fig — E. coli was treated with 550 μM (100 μg/mL) fosmidomycin for one hour at either 37°C (■) or 0°C (■) in either M9-glucose or CAMHB medium. Intracellular fosmidomycin accumulation was monitored by LC-MS (Q-TOF method). (n = 3, error bars represent standard error, p-values were calculated using an unpaired, 2-sample t-test). (TIF) [file pone.0197638.s006.TIF]

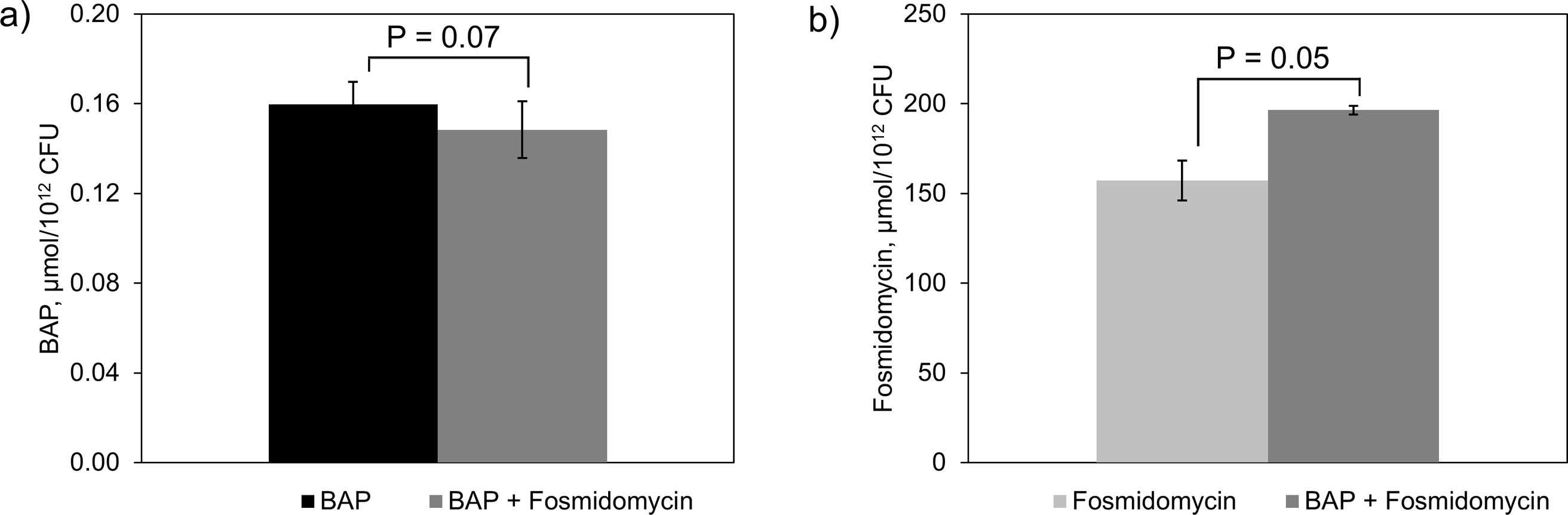

Supplement: S7 Fig — E. coli was treated with 550 μM (100 μg/mL) fosmidomycin, 1250 μM (230 μg/mL) BAP, or both for one hour in CAMHB growth medium. Intracellular BAP (a) and fosmidomycin (b) accumulation was monitored by LC-MS (Q-TOF method). (n = 3, error bars represent standard error, p-values above charts were calculated using an unpaired, 2-sample t-test). (TIF) [file pone.0197638.s007.tif]

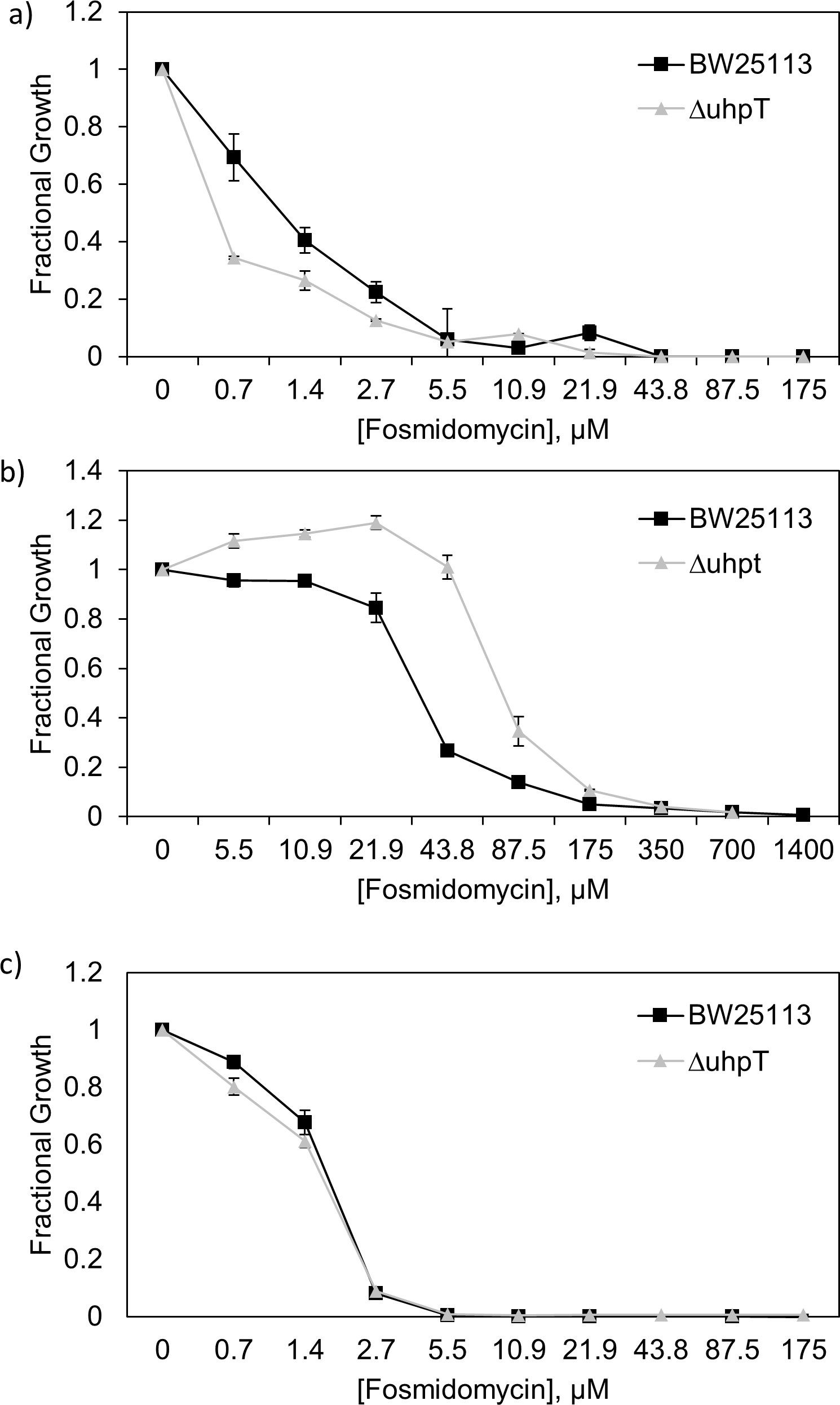

Supplement: S8 Fig — UhpT transporter-containing (■ BW25113) and deficient (▲ ΔuhpT BW25113) E. coli strains were treated with fosmidomycin in CAMHB (a), M9-glucose (b), and M9-glycerol (c) growth medium in biological triplicate. Deletion of UhpT does not significantly impact susceptibility to fosmidomycin. (TIF) [file pone.0197638.s008.TIF]
